# Exploring undergraduate students achievement emotions during ward round simulation: a mixed-method study

**DOI:** 10.1186/s12909-019-1753-1

**Published:** 2019-08-22

**Authors:** Claudia C. Behrens, Diana H. Dolmans, Gerard J. Gormley, Erik W. Driessen

**Affiliations:** 1 Medical Education Unit, Universidad Católica del Norte, Coquimbo, Larrondo, 1281 Coquimbo, Chile; 2 School of Health Professions Education, Maastricht University, Universiteitssingel 60, 6229 ER Maastricht, The Netherlands; 3 Centre for Medical Education, Queen’s University Belfast, University Road, Belfast, BT7 1NN Northern Ireland

**Keywords:** Emotions, Complex simulation, Undergraduate medical students

## Abstract

**Background:**

Simulation based learning (SBL) has increased in its use to best equip students for clinical practice. Simulations that mirror the complex realities of clinical practice have the potential to induce a range of emotions, without a clear understanding of their impact on learning and the learner. Students’ emotional states have important effects on their learning process that can be either positive or negative, and are often difficult to predict. We aimed to determine: (1) To what extent achievement emotions are experienced by medical students during a complex simulation based learning activity, i.e. a ward round simulation (WRS). (2) What their performance scores are and too which extent performance scores do correlate with emotions and 3) how these emotions are perceived to impact learning.

**Methods:**

A mixed methods approach was used in this study. Using an Achievement Emotion Questionnaire, we explored undergraduate medical student’s emotions as they participated in a complex ward round-based simulation. Their performance was rated using an observational ward round assessment tool and correlated with emotions scores. Six focus groups were conducted to provide a deeper understanding of their emotional and learning experiences.

**Results:**

Students experienced a range of emotions during the simulation, they felt proud, enjoyed the simulation and performed well. Students felt proud because they could show in the complex simulation what they had learned so far. Students reported moderate levels of anxiety and low levels of frustration and shame. We found non-significant correlations between achievement emotions and performance during ward round simulation.

**Conclusions:**

Placing undergraduate students in high complex simulations that they can handle raises positive academic achievement emotions which seem to support students’ learning and motivation.

**Electronic supplementary material:**

The online version of this article (10.1186/s12909-019-1753-1) contains supplementary material, which is available to authorized users.

## Background

Medical students and doctors are exposed to a wide variety of feelings and emotions during the course of their clinical practice. Those emotional states can not only influence clinical performance, but also provide motivation to learn and solve complex clinical situations [1, 2]. Simulation has been widely used as a learning tool in healthcare professions. Increasingly, simulated based learning activities have become more complex in order to best prepare medical students for the increasing demands and challenges of modern clinical practice [3]. However, this complexity in simulation-based education may evoke a range of different emotions in learners. It is yet not clear how those emotions impact on learning, well-being and motivation. There is a risk that some of these emotions may well have a negative impact.

Drawing upon neuro and cognitive sciences, emotions appear to modulate perception, memory, attention, reasoning and the capability of transferring learning into new situations [4, 5]. This implies that the connections between emotions and subsequent learning, and performance outcomes are complex. The control value theory of achievement emotions can help to understand the role of emotions in education [6]. Achievement emotions are defined as *“emotions tied directly to achievement activities or achievement outcomes”*. Control-value theory, groups achievement emotions by their valence *(positive* vs. *negative)*, degree of activation *(activating* vs. *deactivating)* and object focus *(activity* vs. *outcome).* Using these dimensions, the theory proposes a three dimensional taxonomy of achievement emotions *(Valance, activation and object)* see Table 1. As example, boredom experienced during a dull simulation session would be considered a negative, deactivating, activity-related achievement emotion; whereas the pride associated with arriving at a correct diagnosis with a challenging patient presentation would be considered a positive, activating, outcome-related achievement emotion.

**Table 1:** A three dimensional taxonomy of achievement emotions (adapted of Pekrun and Stephens 2010)

	Positive (or pleasant)		Negative (or unpleasant)	
Object focus	Activating	Deactivating	Activating	Deactivating
Activity	Enjoyment	Relaxation	Anxiety Anger Frustration	Boredom
Outcome	Hope Joy Pride Gratitude	Relief Contentment	Anxiety Anger Shame	Hopelessness Sadness Disappointment

The control value theory explains the relation between achievement emotions and cognitive processes, as well as motivation. It points out that deactivating emotions may be more detrimental for learning due to the tendency to encourage disconnection from the learning activity [7].

Simulation based education has become more complex in order to prepare medical students for clinical practice, but it has the potential to provoke strong emotional states in medical students**.** Ward Round Simulation (WRS) has emerged, aiming to prepare students for ward round based activities.

Although there is a growing discussion concerning the importance of integrating emotional elements into simulation, particularly negative high arousal ones [8, 9], there is little empirical evidence of the interplay between emotions, performance and learning. Most studies in this area have focused on anxiety and how emotions can influence cognitive load associated with the task. Mills et al. 2016 researched [10] the effect of social evaluation anxiety in performance during simulation-based scenarios, by manipulating the number of other people present with the student during the simulation. They found an association between anxiety and poor performance. Research conducted by Fraser et al. has focused on emotions and cognitive load. They showed that emotions increase cognitive load and reduce learning outcomes [11, 12]. Other studies demonstrated that physical environment in complex simulation can be a source of stress and distraction, which can generate extraneous cognitive load for junior students and probably hinder learning [13]. The conditions under which positive emotions facilitate or suppress learning are unclear, but it has been suggested that all emotions generate an extraneous cognitive load and thus the effect of positive emotions may depend upon their interactions with other sources of cognitive load [12].

Further research in this area is necessary to better understand the role of emotions during a complex simulation and how these emotions affect the learning process.

This study aims to determine (1) to what extent achievement emotions are experienced by medical students during a complex simulation based learning activity, i.e. a ward round simulation (WRS). (2) What their performance scores are and to which extent performance scores do correlate with emotions and 3) how these emotions are perceived to impact their learning.

## Methods

This study used an explanatory sequential mixed method design [14] in order to answer the research questions. The emotions experienced during the exercise were studied through the achievement emotions questionnaire (AEQ) Spanish version [15] and students’ performance in WRS were rated using the Postgraduate Ward Round Simulation assessment tool (PgWRS) [16]. Focus groups were conducted to provide a deeper understanding of the role of these emotions on their learning process. The Reporting Guidelines for Health Care Simulation Research were used [17].

### Setting

The study was carried out in the Clinical Simulation Center at Universidad Católica del Norte, Chile. The medical degree program lasts 7 years. Students start clinical practice in year 3.The last 2 years correspond to internships where students are enrolled in emergency ward round as part of their medical training. At this stage of training, students are expect to be able to clinically assess patients, provide a provisional diagnosis, determine a management plan and develop their ward based skills. Students have clinical exposure and simulation-based learning activities throughout the curriculum. Between 50 and 65 students annually enroll in the program.

### Intervention

Each participant took part in an emergency ward round simulation. Characteristics of WRS consist of complex clinical scenarios situated in a simulated clinical ward that involves multiple elements such as managing more than one patient, interacting with relatives and other healthcare professionals, and dealing with multiple competing tasks activities where interruptions and distractions occur [3, 18–20].

Briefing was conducted in order to orientate participants before the simulation. During the WRS, each participant was involved on a “hand over” exercise for 25 min followed by a 45 min debriefing using a “Debriefing with good judgment” approach [21]. This approach utilizes a self-reflection process that helps students recognize and resolve clinical and behavioral dilemmas raised by the simulation itself and the instructor. This supports the participant to critically reflect on their emotions, actions and how they could modify future performance.

In this emergency simulated ward, participants had to attend 4 simulated patients, two of them were accompanied by relatives. Situations with different emotional grades into scenarios were promoted: 1) a patient being very grateful after being treated for a supraventricular tachycardia (a form of heart arrhythmia); 2) family conflict regarding the cardiorespiratory arrest (secondary to asphyxia) in an infant 3) an unconscious patient brought to the emergency room by a healthcare provider after a motor vehicle accident and 4) a patient who is having a miscarriage.

A qualified nurse was part of the scenarios as well as a doctor from the staff who received the patients at the change of shift after they were taken care by the participants. Each role player had to adhere to scenario scripts that guided their performance and roles in the scenarios. They were trained by one of the researchers (CP) who has experience in training simulated patients. During the briefing, each participant was asked to consider the activity as if it was their first day as a ‘junior doctor’ working in a clinical ward. The task was to gain an overview of the patients’ cases, related patient files and patient medication charts. They had to define consultation goals, conduct the ward round and re-evaluate the patients’ therapy.

### Recruitment and sampling

For this study, all 6th year medical students (*n* = 55) were invited by email to participate in the study. Students had some prior experience of simulation-based learning activities, but this was the first time they faced a WRS exercise.

This study received approval from the Research Ethics Committee of Universidad Católica del Norte (F.M: 82–2017) and informed written consent was obtained from all the participants.

### Quantitative data collection and analysis

#### Achievement emotions questionnaire (AEQ)

AEQ is a multidimensional self-report instrument developed to measure the emotions of students in academic situations and was used to identify students’ achievement emotions before, during and after simulation [22–24]. The Spanish version [15] was applied immediately after debriefing (Additional file 1). This questionnaire includes 68 items that measure eight emotions: enjoyment, hope, pride, anger, anxiety, shame, hopelessness, and boredom. Students rate their emotional experiences on a five point Likert scale from ‘strongly disagree’ (1) – ‘strongly agree’ (5). A score of 4 or higher was considered high/good, between 2 and 3 quite neutral and 2 or less, low.

#### Postgraduate Ward round simulation assessment tool (PgWRS)

A validated Postgraduate Ward Round Simulation assessment tool (PgWRS) [3, 16] was used to rate participants’ performances (Additional file 2).

This tool assesses 9 domains: Task management, clinical skills, acutely ill patients, prescribing techniques, response to interruptions, written documentation, communication, health and safety and professionalism. For each domain, a five-point Likert scale was used to assess domain performance, ranging from ‘1’ (very poor performance) to ‘5’ (outstanding performance). Because of the length of the exercise, written documentation was not assessed. A score of 4 or higher was considered good performance, between 2 and 3 regular performance, need to improve and 2 or less, low performance.

All simulation sessions were video recorded. Video recordings were viewed by two independent raters who were experienced medical doctors and educators and have received training in using PgWRS. Thirty five out of fifty-three participants (66%) were scored by the second rater. Ratings of the two observers had a high correlation (*r* = 0.792, *p* < 0.001).

Descriptive statistics were calculated and AEQ scores were averaged. The Cronbach’s alpha’s of the Emotion indexes were calculated. Performance assessment scores were summarized and correlated with the AEQ scores using Pearson coefficient. SPSS version 23 was used for the calculations.

### Qualitative data collection and analysis: focus groups

After the simulation exercise, all participants took part in six different focus groups of 8–9 medical students. The focus groups were carried out in order to provide a deeper understanding of how medical students describe their emotional experiences during the WRS and the impact of these emotions on their learning. One author (CBP) facilitated the focus groups. Each focus group lasted between 35 and 45 min. Questions addressed students’ emotions during simulation and the role of such emotions in their learning process. (The interview guide is available in Additional file 3). Focus group interviews were audio-recorded and transcribed verbatim.

Template analysis approach for the data analysis was used [25]. A succession of coding templates consisting of hierarchically structured themes was carried out and iteratively applied to the data. The themes were modified continuously as the analysis progressed. To start with, the research anticipated some themes to be identified in the analysis. One author and a research assistant independently coded 2 focus group transcripts. The research team discussed and reviewed the emergent themes based on importance and relevance to the study. Final agreement was achieved on themes. After the agreement, the initial coding template was developed and applied to all focus group transcripts. The research team met regularly during the study to discuss the analysis.

## Results

Fifty-three out of fifty-five subjects (96.4%) participated in the study, with an equal ratio of male to female trainees with a mean age of 23.8 years old.

### Quantitative results

#### Achievement emotions experienced by students

The positive emotions obtained the highest scores, being “enjoyment”, “pride” and “hope” scored with a mean of 4.08 (SD = 0.59), 3.84 (SD = 0.75) and 3.57 (SD = 0.56) respectively; whereas negative emotions had neutral scores, being anxiety rated with 2.89 (SD = 0.77) and boredom with 1.09 (SD = 0.24), scale 1–5. Table 2 provides an overview of the descriptive statistics with its frequency distribution of students’ responses. Additional file 4 provides the statistics for each item that composes the achievement emotions. There was no statistical differences between emotions before, during and after simulation. We, therefore, used the mean scores across the three measurement moments, a so called overall score. The reliability of overall scale was high (α = 0.820). However, those values for anger, hopelessness and boredom were lower due to the poor variance on students’ responses for those emotions, as shown in Table 2.

**Table 2:** Frequency distribution of achievement emotions experienced by participants in ward round simulation exercise (*N* = 53)

	Score 1%	Score 2%	Score 3%	Score 4%	Score 5%	Overall (1–5)	SD	α
Enjoyment	0	0	15,1	52,8	32	4.08	0.59	0.76
Hope	0	3,8	45,3	45,3	5,7	3.57	0.56	0.72
Pride	0	5,7	18,9	56,5	19	3.84	0.75	0.81
Anger	83	13,2	3,77	0	0	1.29	0.38	0.51
Anxiety	3,8	22,6	47,2	24,5	1,9	2.98	0.77	0.86
Shame	32	43,4	18,9	5,68	0	1.97	0.84	0.88
Hopelessness	79	20,8	0	0	0	1.22	0.30	0.62
Boredom	89	11,3	0	0	0	1.09	0.24	0.62

Scores values expressed as percentage, overall as mean + SD and Cronbach alpha

#### Students’ performance

For the four patient scenarios, the overall score reached by participants had a mean of 4.26 out of 5 (SD + 0.43), being the pass score of 4. Its results per component are shown in Additional file 5. The best domain-specific learning outcomes that were attained were “communication with colleagues” with a mean of 4.58 + 0.63, “communication with patients/relatives” with 4.58 + 0.71; whereas the lowest outcome was “prescribing techniques” with 3.92 + 0.87.

All achievement emotions measured by the questionnaire had statistically non-significant correlations with performance (see Table 3).

**Table 3:** Correlation between WRS performance scores and Achievement emotions experienced by participants (*N* = 53)

	Pearson Coefficient	*p value*
Enjoyment	−0.108	0.441
Hope	0.044	0.756
Pride	−0.013	0.927
Anger	−0.254	0.067
Anxiety	−0.227	0.102
Shame	−0.196	0.159
Hopelessness	−0.223	0.108
Boredom	−0.244	0.079

Coefficient of correlation Pearson

### Qualitative results

Three major themes emerged from the analysis. Feelings of enjoyment and pride during the experience motivated further study because appropriate clinical outcomes were perceived as a reward to their effort and encouraged further study. Furthermore, students mentioned that anxiety during the exercise could potentially be beneficial for their learning because it made them aware of their learning needs. Negative emotions as frustration and shame were seen as detrimental for their learning because these affect their self-esteem and reduce motivation for studying.

#### ‘Pride in my learning’

Participants reported a range of positive emotions during the simulation. Feelings of pride, satisfaction and happiness were the most common after patients responded positively to their intervention. Students felt proud and enjoyed it because they could show what they have learned. Those emotions generated a suitable condition for learning and it promoted discussion during debriefing.

*“Satisfaction of my performance, encourages me to study even more, because I am seeing good results” (Student #51)*

*“It was great when the patient’s blood pressure started to rise, I felt so motivated and happy … .I realized that I have important concepts about how to manage critical patients” (Student #31).*

#### ‘Worrying to get better’

Most participants reported moderate degrees of anxiety before and during the exercise due to the fear of not performing well and the uncertainty of what would occur during the simulation. Making decisions during emergency situations was new and challenging for the students. However, the moderate anxiety levels experienced by them and their worries about being insufficiently prepared was considered by the participants as a positive promoter of learning. It reminded them the necessity to be better prepared to face emergency situations and the need to adopt a deep approach for study.

*“I do not like to feel nervous and anxious because that reflects that I did not study enough, it makes me feel insecure in my decision making. So, for the next one, I will have to study harder to feel that I am doing it well.” (Student #24)*

*“The frustration that I felt because I could not manage the patient will make me study a lot, not only for the assessment, this can’t happen again.” (Student #5)*

#### ‘Well … that didn’t go well!’

Shame and frustration were negative emotions experienced by participants during and after simulation. Those negative feelings appeared after failing to manage adequately life-threatening conditions. They reported that feeling frustrated and embarrassed negatively impacted learning because it reduced their interest and motivation for studying. Those negative emotions impacted the performance during the simulation and induced to make wrong decisions. Finally, these emotions affected their self-esteem.

*“The negative emotions do not permit me to remember anything. I felt very anxious and frustrated during the exercise. Probably I will not remember anything that we talked about tomorrow because I am sad, disappointed and this affects my self-esteem … . I usually study a lot!...” (Student #8)*

A small group of participants experienced shame during the simulation, which seemed to be a strong external motivation for learning. They referred that these unpleasant feelings makes them study harder in order to not experience it again and demonstrate their clinical skills in a new scenario.

## Discussion

In this study, students, who performed well in a ward round simulation, experienced mainly positive academic emotions. Although previous studies have reported deficiencies in ward round skills in undergraduate medical students [3, 19, 26–28], the performance scores in this study were high. The high performance in this group of students could explain that the positive emotion scores were higher in comparison with negative emotions during the simulation. Moreover, the high performance scores possibly caused a ceiling effect, i.e. the performance scores did not correlate with the scores on the achievement emotions questionnaire.

Our qualitative data supports theoretical assumptions of the control value theory [29] about how emotions perceived by students can influence performance in simulation settings. The data suggests that positive emotions as pride, enjoyment and hope create a suitable condition for learning, leading to great interest and encouraging further study. Those emotions can be considered as positive, activating and outcomes-related achievement emotions.

Negative emotions such as anxiety, shame and frustration were also found. Although these scores were quite neutral or low, qualitative data suggested that these could bring different consequences for learning. In this context, they can act as either as a promoter or inhibitor of learning. Moderate feelings of anxiety, although negative, were perceived to have a positive effect on learning, because students felt they needed to prepare better and encouraged further study. This implies that moderate feelings of anxiety may act as a positive promoter of learning instead of a negative drive as specified according to the control value theory [6]. Nevertheless, it might be possible that a high level of anxiety might have negative effects on learning. Feeling of frustration and shame, although, they scored rather low, were perceived to reduce students’ interest and motivation for studying, hampered their decision making process and negatively affected their self-esteem. These latter findings are consistent with the academic achievement emotions literature where negative emotions are associated to poor intrinsic motivation, the use of superficial processing strategies and reduced interest for learning [5, 23, 29]. Notwithstanding, for a group of students, shame is considered as a strong external motivation for studying which is concordant with theoretical researches in emotions and performance [29].

## Limitations

This study had several limitations. The study was conducted in a single center and we used a single intervention which makes that we have to be careful with generalizing our results to other contexts. A long-term follow up would have provided us with more information about the impact of the emotions experienced during the simulations. Another limitation is that we studied perceived influence of emotions on study behavior and motivation, and not the actual impact. A potential limitation was a possible selection bias, because participants were self-selected volunteers. However, 96.4% of the students cohort participated in the study. More research is needed to explore the relationship between emotions and learning in less experienced cohort of students.

## Conclusions

Placing students in high complex simulations in which they perform well raises positive academic achievement emotions from the students’ perceptions, such as enjoyment and pride and moderate negative academic achievement emotions, such as anxiety. The positive emotions of pride and enjoyment were perceived by students as a positive drive for learning. The same holds for a moderate level of anxiety, given that it makes students realize they need to prepare better for dealing with these complex problems. Shame, which scored rather low, was perceived to be detrimental for learning, because it is perceived to negatively impact students’ self-esteem. Emotions did not correlate significantly with performance, probably because students had a high performance score.

These results highlight the importance of incorporating emotions in instructional design of complex simulation based learning activities, which is suitable to implement in undergraduate curriculum in order to optimize student’s learning process.

## Additional files

Additional file 1: Achievement emotions questionnaire in Spanish version, which was used to collect quantitative data about emotions. English version is also included. (DOCX 34 kb)

Additional file 2: Postgraduate Ward round simulation assessment tool, which was used to rate students’ performance. (DOCX 15 kb)

Additional file 3: Focus group guide, which was used to collect qualitative data. (DOCX 15 kb)

Additional file 4: Achievement Emotions Questionnaire results by scale and items (DOCX 20 kb)

Additional file 5: Ward round simulation score (PgWRE) results, with individual components (DOCX 15 kb)

## Data Availability

The datasets used and/or analyzed during the current study are available from the corresponding author on reasonable request.

## Abbreviations

AEQ: Achievement Emotions Questionnaire
PgWRS: Postgraduate Ward Round Simulation
SBL: Simulation based learning
WRS: Ward Round Simulation
