# Supplementary material for: Exploring undergraduate students achievement emotions during ward round simulation: a mixed-method study
Source: BMC Med Educ. 2019 Aug 22;19:316. doi: 10.1186/s12909-019-1753-1 (PMC6704623; doi:10.1186/s12909-019-1753-1)
Supplement: Supplementary file 1 — Achievement emotions questionnaire in Spanish version, which was used to collect quantitative data about emotions. English version is also included. (DOCX 34 kb) [file 12909_2019_1753_MOESM1_ESM.docx]

Additional file 1: **Concurrent assessment of state achievement emotions (for class-related emotions, before, during and after the class) English version ^24^.**

**INSTRUCTIONS**

Attending simulation lessons at university can induce different feelings. This part of the questionnaire refers to emotions you may experience when being in simulation lessons at university. Before answering the questions on the following pages, please recall some typical situations of being in simulation which you have experienced during the course of your studies. Read each item carefully and respond using the scale provided (1) strongly disagree to (5) Strongly agree.

**(1) BEFORE SIMULATION**

The following questions pertain to feelings you may experience before being in simulation. Please indicate how you feel, typically, before you go to simulation.

| Item |  | 1 | 2 | 3 | 4 | 5 | NA |
| --- | --- | --- | --- | --- | --- | --- | --- |
| CJOA1B | I get excited about going to simulation |  |  |  |  |  |  |
| CJOC1B | I am looking forward to learning a lot in simulation |  |  |  |  |  |  |
| CJOM1B | I am motivated to go to this class because it is exciting. |  |  |  |  |  |  |
| CHOA1B | I am confident when I go to class |  |  |  |  |  |  |
| CHOC1B | I am optimistic that I will be able to keep up with the material |  |  |  |  |  |  |
| CHOM1B | Being confident that I will understand the material motivates me |  |  |  |  |  |  |
| CAGM1B | I wish I did not have to attend class because it makes me angry |  |  |  |  |  |  |
| CHOA2B | I am full of hope |  |  |  |  |  |  |
| CHOM2B | My confidence motivates me to prepare for class |  |  |  |  |  |  |
| CHOM3B | My hopes that I will be successful motivate me to invest a lot of effort |  |  |  |  |  |  |
| CAXA1B | Thinking about the class makes me feel uneasy |  |  |  |  |  |  |
| CAXA2B | I feel scared |  |  |  |  |  |  |
| CAXC1B | Even before class, I worry whether I will be able to understand the material |  |  |  |  |  |  |
| CAXC2B | I worry whether I am sufficiently prepared for the lesson |  |  |  |  |  |  |
| CAXC3B | I worry whether the demands might be too great |  |  |  |  |  |  |
| CAXM1B | Because I am so nervous I would rather skip the class |  |  |  |  |  |  |
| CHLA1B | The thought of this class makes me feel hopeless |  |  |  |  |  |  |
| CHLC1B | Even before class, I am resigned to the fact that I won´t understand the material |  |  |  |  |  |  |
| CHLM1B | Because I´ve given up, I don´t have energy to go to class |  |  |  |  |  |  |

**(2) DURING SIMULATION**

The following questions pertain to feelings you may experience during simulation. Please indicate how you feel, typically, during simulation leassons.

|  |  | 1 | 2 | 3 | 4 | 5 | NA |
| --- | --- | --- | --- | --- | --- | --- | --- |
| CJOA2D | I enjoy being in class |  |  |  |  |  |  |
| CAGC2D | Thinking about all the useless things I have to learn makes me irritated |  |  |  |  |  |  |
| CPRC1D | I take pride in being able to keep up with the material |  |  |  |  |  |  |
| CPRM1D | When I make good contributions in class, I get even more motivated |  |  |  |  |  |  |
| CPRP1D | When I do well in class, my heart throbs with pride. |  |  |  |  |  |  |
| CAGA1D | I feel frustrated in class |  |  |  |  |  |  |
| CAGC1D | Thinking about the poor quality of the course makes me angry |  |  |  |  |  |  |
| CJOM3D | It is so exciting that I could sit in class for hours listening to the professor |  |  |  |  |  |  |
| CHOC3D | I am confident because I understand the material |  |  |  |  |  |  |
| CAXA3D | I feel nervous in class |  |  |  |  |  |  |
| CAXC4D | I worry the others will understand more than me |  |  |  |  |  |  |
| CAXM2D | I get scared that I might say something wrong, so I´d rather not say anything |  |  |  |  |  |  |
| CAXP2D | I get tense in class |  |  |  |  |  |  |
| CAXP3D | When I do not understand something important in class, my heart races |  |  |  |  |  |  |
| CSHA2D | I am ashamed |  |  |  |  |  |  |
| CSHC1D | If the others knew that I don´t understand the material I would be embarrassed |  |  |  |  |  |  |
| CSHC2D | When I say anything in class I feel like I am making a fool of myself |  |  |  |  |  |  |
| CSHC3D | I am embarrassed that I can´t express myself well |  |  |  |  |  |  |
| CSHM1D | After I said something in class I wish I could crawl into a hole and hide |  |  |  |  |  |  |
| CSHP1D | When I say something in class I feel like I turn red |  |  |  |  |  |  |
| CSHP2D | Because I get embarrassed, I become tense and inhibited |  |  |  |  |  |  |
| CSHP3D | When I talk in class start stuttering |  |  |  |  |  |  |
| CHLA2D | I feel hopeless. |  |  |  |  |  |  |
| CHLC2D | I have lost all hope in understanding this class |  |  |  |  |  |  |
| CHLP1D | Because I don´t understand the material I look disconnected and resigned |  |  |  |  |  |  |
| CBOA1D | I get bored |  |  |  |  |  |  |
| CBOC2D | Because I get bored my mind begins to wander |  |  |  |  |  |  |
| CBOM1D | I am tempted to walk out of the lecture because it is so boring |  |  |  |  |  |  |
| CBOM2D | I think about what else I might be doing rather than sitting in this boring class |  |  |  |  |  |  |
| CBOM3D | Because the time drags I frequently look at my watch |  |  |  |  |  |  |
| CBOP1D | I get so bored I have problems staying alert |  |  |  |  |  |  |
| CBOP2D | I get restless because I can´t wait for the class to end |  |  |  |  |  |  |
| CBOP3D | During the class I feel like could sink into my chair |  |  |  |  |  |  |
| CBOP4D | I start yawing in class because I am so bored |  |  |  |  |  |  |

**(3) AFTER SIMULATION (DEBRIEFING)**

The following questions pertain to feelings you may experience after being in simulation. Please indicate how you feel, typically, after you go to simulation.

|  |  | 1 | 2 | 3 | 4 | 5 | NA |
| --- | --- | --- | --- | --- | --- | --- | --- |
| CJOA3A | After class I start looking forward to the next class |  |  |  |  |  |  |
| CJOC3A | I am glad that it paid off to go to class |  |  |  |  |  |  |
| CPRC3A | I think that I can be proud of what I know about this subject |  |  |  |  |  |  |
| CPRC4A | I am proud of the contributions I have made in class |  |  |  |  |  |  |
| CPRM3A | I would like to tell my friends about how well I did in this course |  |  |  |  |  |  |
| CPRA1A | I am proud of myself |  |  |  |  |  |  |
| CJOC2A | I am happy that I understood the material |  |  |  |  |  |  |
| CPRM2A | Because I take pride in my accomplishments in this course, I am motivated to continue |  |  |  |  |  |  |
| CAGA2A | I am angry |  |  |  |  |  |  |
| CAGC3A | When I think of the time I waste in class I get aggravated. |  |  |  |  |  |  |
| CAGM2A | I wish I could tell the teachers off |  |  |  |  |  |  |
| CSHC4A | I am ashamed because others understood more of lecture that I did |  |  |  |  |  |  |
| CHSM2A | I´d rather not tell anyone when I don´t understand something in class |  |  |  |  |  |  |
| CHLC3A | I feel hopeless continuing in this program of study |  |  |  |  |  |  |
| CHLP2A | I feel so hopeless all my energy is depleted |  |  |  |  |  |  |

**Concurrent assessment of state achievement emotions (for class-related emotions, before, during and after the class). Spanish version ^14^.**

**INSTRUCCIONES**

"Asistir a talleres de simulación puede provocar diferentes sentimientos. Esta parte del cuestionario hace referencia a las emociones que puedes experimentar mientras estás en talleres de simulación. Lee cuidadosamente y responde indicando la frecuencia con la que experimentas aquello que describe cada ítem en una escala de 1 (nunca) a 5 (siempre)".

**(1) ANTES DE LA SIMULACIÓN**

"Los siguientes ítems se refieren a los sentimientos que puedes experimentar ANTES de estar en simulación. Indica cómo te sientes generalmente antes de ir a simulación."

| Item |  | 1 | 2 | 3 | 4 | 5 | NA |
| --- | --- | --- | --- | --- | --- | --- | --- |
| CJOA1B | Me entusiasma ir a simulación. |  |  |  |  |  |  |
| CJOC1B | Tengo ganas de aprender mucho en esta simulación. |  |  |  |  |  |  |
| CJOM1B | Estoy motivado/a a ir a esta simulación porque es interesante. |  |  |  |  |  |  |
| CHOA1B | Me siento seguro/a de mí mismo/a cuando voy a simulación. |  |  |  |  |  |  |
| CHOC1B | Confío en que podré llevar el caso estudiado. |  |  |  |  |  |  |
| CHOM1B | Me motiva el estar seguro/a de que entenderé el material. |  |  |  |  |  |  |
| CAGM1B | Quisiera no tener que asistir a simulación porque me enoja. |  |  |  |  |  |  |
| CHOA2B | Me siento lleno/a de esperanzas. |  |  |  |  |  |  |
| CHOM2B | Mi seguridad me motiva a prepararme para la simulación. |  |  |  |  |  |  |
| CHOM3B | La esperanza de obtener buenos resultados me motiva a esforzarme mucho. |  |  |  |  |  |  |
| CAXA1B | Pensar sobre la simulación me hace sentir preocupado/a. |  |  |  |  |  |  |
| CAXA2B | Tengo miedo. |  |  |  |  |  |  |
| CAXC1B | Incluso antes de la simulación me preocupa si podré entender la discusión y el caso. |  |  |  |  |  |  |
| CAXC2B | Me preocupa si estaré lo suficientemente preparado/a para la simulación. |  |  |  |  |  |  |
| CAXC3B | Me preocupa que el nivel de exigencia pueda ser demasiado alto. |  |  |  |  |  |  |
| CAXM1B | Como estoy tan nervioso/a prefiero faltar a la simulación. |  |  |  |  |  |  |
| CHLA1B | Pensar en esta simulación me hace sentir desesperanzado/a. |  |  |  |  |  |  |
| CHLC1B | Incluso antes de la simulación estoy resignado/a a que no entenderé el caso y la discusión. |  |  |  |  |  |  |
| CHLM1B | Como ya me he dado por vencido/a, no tengo ganas de ir a simulación. |  |  |  |  |  |  |

**(2) DURANTE LA SIMULACIÓN**

"Los siguientes ítems se refieren a los sentimientos que puedes experimentar DURANTE la simulación. Indica cómo te sientes generalmente durante la simulación."

|  |  | 1 | 2 | 3 | 4 | 5 | NA |
| --- | --- | --- | --- | --- | --- | --- | --- |
| CJOA2D | Disfruto de estar en simulación. |  |  |  |  |  |  |
| CAGC2D | Me irrita pensar en todas las cosas inútiles que tengo que aprender. |  |  |  |  |  |  |
| CPRC1D | Me enorgullece poder llevar al día los temas del taller. |  |  |  |  |  |  |
| CPRM1D | Cuando hago buenos aportes en simulación me siento más motivado/a. |  |  |  |  |  |  |
| CPRP1D | Cuando me va bien en simulación mi corazón late con orgullo. |  |  |  |  |  |  |
| CAGA1D | Me siento frustrado/a en simulación. |  |  |  |  |  |  |
| CAGC1D | Pensar en lo bajo que es el nivel de la materia me hace enojar. |  |  |  |  |  |  |
| CJOM3D | Me entusiasma tanto esta simulación que podría pasar horas escuchando la discusión. |  |  |  |  |  |  |
| CHOC3D | Me siento seguro/a porque comprendo el caso. |  |  |  |  |  |  |
| CAXA3D | Estoy nervioso/a en simulación. |  |  |  |  |  |  |
| CAXC4D | Me preocupa que los demás vayan a entender más que yo. |  |  |  |  |  |  |
| CAXM2D | Me da miedo equivocarme, así que mejor no digo nada. |  |  |  |  |  |  |
| CAXP2D | Me pongo tenso/a en simulación. |  |  |  |  |  |  |
| CAXP3D | Cuando no entiendo algo importante en simulación se me acelera el corazón. |  |  |  |  |  |  |
| CSHA2D | Me siento avergonzado/a. |  |  |  |  |  |  |
| CSHC1D | Me daría vergüenza que los demás supieran que no entiendo el material. |  |  |  |  |  |  |
| CSHC2D | Cuando digo algo en simulación siento que estoy haciendo el ridículo. |  |  |  |  |  |  |
| CSHC3D | Me da vergüenza no saber expresarme bien. |  |  |  |  |  |  |
| CSHM1D | Después de haber dicho algo en simulación quisiera que me tragara la tierra. |  |  |  |  |  |  |
| CSHP1D | Cuando digo algo en simulación siento que me pongo colorado/a. |  |  |  |  |  |  |
| CSHP2D | La vergüenza me pone tenso/a y me hace sentir inhibido/a. |  |  |  |  |  |  |
| CSHP3D | Cuando hablo en simulación empiezo a tartamudear. |  |  |  |  |  |  |
| CHLA2D | Me siento desesperanzado/a. |  |  |  |  |  |  |
| CHLC2D | He perdido todas las esperanzas de entender en esta simulación. |  |  |  |  |  |  |
| CHLP1D | Como no entiendo el material se me ve desconectado/a y resignado/a. |  |  |  |  |  |  |
| CBOA1D | Me aburro. |  |  |  |  |  |  |
| CBOC2D | Como me aburro mi imaginación vuela. |  |  |  |  |  |  |
| CBOM1D | La simulación es tan aburrida que tengo ganas de irme. |  |  |  |  |  |  |
| CBOM2D | Pienso en qué más podría estar haciendo en vez de estar sentado/a en esta simulación aburrida. |  |  |  |  |  |  |
| CBOM3D | Miro el reloj a cada rato porque el tiempo pasa muy lentamente. |  |  |  |  |  |  |
| CBOP1D | Me aburro tanto que me cuesta permanecer despierto/a. |  |  |  |  |  |  |
| CBOP2D | Me impaciento porque no veo la hora de que termine la simulación. |  |  |  |  |  |  |
| CBOP3D | Durante la simulación siento que me duermo. |  |  |  |  |  |  |
| CBOP4D | Comienzo a bostezar en simulación de tan aburrido/a que estoy. |  |  |  |  |  |  |

**(3) DESPUÉS DE LA SIMULACIÓN (DEBRIEFING)**

"Los siguientes ítems se refieren a los sentimientos que puedes experimentar DESPUÉS de la simulación. Indica cómo te sientes generalmente después de la simulación."

|  |  | 1 | 2 | 3 | 4 | 5 | NA |
| --- | --- | --- | --- | --- | --- | --- | --- |
| CJOA3A | Al terminar la simulación ya estoy deseando que llegue la próxima. |  |  |  |  |  |  |
| CJOC3A | Me alegra que haya valido la pena ir a simulación. |  |  |  |  |  |  |
| CPRC3A | Creo que puedo sentirme orgulloso/a de lo que sé sobre esta materia. |  |  |  |  |  |  |
| CPRC4A | Me siento orgulloso/a de los aportes que he hecho en simulación. |  |  |  |  |  |  |
| CPRM3A | Me gustaría contarles a mis amigo/as lo bien que me fue en esta materia. |  |  |  |  |  |  |
| CPRA1A | Estoy orgulloso/a de mí mismo. |  |  |  |  |  |  |
| CJOC2A | Estoy contento/a de haber aprendido la patología(s). |  |  |  |  |  |  |
| CPRM2A | Me siento motivado/a para continuar con esta materia porque me enorgullecen mis logros. |  |  |  |  |  |  |
| CAGA2A | Estoy enojado/a. |  |  |  |  |  |  |
| CAGC3A | Me irrita mucho pensar en el tiempo que pierdo en simulación. |  |  |  |  |  |  |
| CAGM2A | Desearía poder retar a los profesore/as. |  |  |  |  |  |  |
| CSHC4A | Me da vergüenza que otro/as hayan entendido la simulación mejor que yo. |  |  |  |  |  |  |
| CHSM2A | Cuando no entiendo algo en simulación prefiero que nadie se entere. |  |  |  |  |  |  |
| CHLC3A | Siento que en esta carrera nunca me va a ir bien. |  |  |  |  |  |  |
| CHLP2A | Me siento tan desesperanzado/a que no tengo energías. |  |  |  |  |  |  |
