# Supplementary material for: Exploring undergraduate students achievement emotions during ward round simulation: a mixed-method study
Source: BMC Med Educ. 2019 Aug 22;19:316. doi: 10.1186/s12909-019-1753-1 (PMC6704623; doi:10.1186/s12909-019-1753-1)
Supplement: Supplementary file 2 — Postgraduate Ward round simulation assessment tool, which was used to rate students’ performance. (DOCX 15 kb) [file 12909_2019_1753_MOESM2_ESM.docx]

Additional file 2**:** Postgraduate Ward Round Simulation assessment tool (PgWRS) ^15^

| **Task management**  Candidate has a good overview and prioritizes appropriately. Candidate conducts all essential tasks and clinical procedures. Delivers an appropriate handover.  Very poor performance Outstanding performance  1 2 3 4 5 N/A |
| --- |
| **Clinical skills**  Candidate demonstrates effective history taking skills. Candidate demonstrates appropriate examination techniques and initiates appropriate interventions. Candidate interprets results and makes informed decisions.  Very poor performance Outstanding performance  1 2 3 4 5 N/A |
| **Accurately ill patients**  Candidate recognizes and systematically assesses the patient using ABCDE approach. Candidate manages the acutely ill patient appropriately and demonstrates good time management skills, recognizing when to get help from a senior colleague.  Very poor performance Outstanding performance  1 2 3 4 5 N/A |
| **Prescribing technique**  Candidate demonstrates a safe and appropriate prescribing technique.  Very poor performance Outstanding performance  1 2 3 4 5 N/A |
| **Written documentation**  Candidate completes written tasks appropriately.  Very poor performance Outstanding performance  1 2 3 4 5 N/A |
| **Response to interruptions**  Candidate responds appropriately to interruptions and follows up. Candidate responds appropriately and reacts to nursing observations.  Very poor performance Outstanding performance  1 2 3 4 5 N/A |
| **Communication**  Candidate demonstrates good interpersonal skills and uses appropriate language. Candidate responds appropriately to each patients´ care requirement, answering questions and keeping patients informed. Candidate communicates effectively with colleagues.  Very poor performance Outstanding performance  Relationship with patients/relatives  1 2 3 4 5 N/A  Working with colleges  1 2 3 4 5 N/A |
| **Health and safety**  Candidate prevents cross infections and demonstrates safe medical practice during the ward simulation exercise. Safe medical practice can be defined as patient safety, safe disposal of sharps, appropriate use of PPE and maintain patient dignity.  Very poor performance Outstanding performance  1 2 3 4 5 N/A |
| **Professionalism**  Candidate acts in a manner becoming of their actual grade of practice, is polite, considerate and honest. Candidate treats patients with dignity, respecting patients´ privacy and right to confidentiality.  Very poor performance Outstanding performance  1 2 3 4 5 N/A |

| What behaviors does the candidate exhibit during the exercise? |
| --- |
| What are the candidate strengths? |
| What areas does the candidate need to improve? |
| Overall global judgment of performance:  Very poor performance Outstanding performance  1 2 3 4 5 N/A |

My overall global judgment of this candidate is (please circle):

PASS FAIL
