# Supplementary material for: Exploring undergraduate students achievement emotions during ward round simulation: a mixed-method study
Source: BMC Med Educ. 2019 Aug 22;19:316. doi: 10.1186/s12909-019-1753-1 (PMC6704623; doi:10.1186/s12909-019-1753-1)
Supplement: Supplementary file 3 — Focus group guide, which was used to collect qualitative data. (DOCX 15 kb) [file 12909_2019_1753_MOESM3_ESM.docx]

Additional file 3**:** Focus group questions

**How do medical students describe their subjective lived experiences**

1. **such emotions during a simulation based learning activity?**
2. **and the impact of these emotions on their learning?**

**Introductory question:**

1. *Introduce yourself*

*-      Name:*

*-      Internship by which they are rotating*

**Transition question:**

1. *Can you describe how you felt during the simulation activity?*

**Key questions:**

We will talk about emotions in medical education. Every day, we experience emotions with our patients which can influence not only our clinical performance, but also our motivation and ability to learn and solve complex clinical problems

1. *What emotions did you feel during simulation activity? How can you describe them? (Consider briefing, simulation and debriefing)*
2. *What event or situation caused those emotions? Can you explain that further?*

When we talk about learning on simulation, we mean how this activity will lead to change your performance in your clinical practice

1. *What did you learn during the simulation?*
2. *Which role did emotions play in your learning?*

**Wrap-up question:**

1. *In sum (…). Would you like to address aspects which are not discussed yet?*
