# Supplementary material for: Exploring undergraduate students achievement emotions during ward round simulation: a mixed-method study
Source: BMC Med Educ. 2019 Aug 22;19:316. doi: 10.1186/s12909-019-1753-1 (PMC6704623; doi:10.1186/s12909-019-1753-1)
Supplement: Supplementary file 4 — Achievement Emotions Questionnaire results by scale and items (DOCX 20 kb) [file 12909_2019_1753_MOESM4_ESM.docx]

Additional file 4**:** Achievement Emotions Questionnaire results by scale and items

| **Enjoyment Scale statistics: Mean=4.08; SD=0.59; α=0.76** | | |
| --- | --- | --- |
|  | Mean | SD |
| I get excited about going to class | 3.85 | 1.09 |
| I enjoy being in class | 3.81 | 1.08 |
| After class I start looking forward to the next class | 3.96 | 0.97 |
| I am looking forward to learning a lot in this class | 4.55 | 0.86 |
| I am happy that I understood the material | 4.41 | 0.75 |
| I am glad that it paid off to go to class | 4.86 | 0.40 |
| I am motivated to go to this class because it is exciting | 4.30 | 0.88 |
| It is so exciting that I could sit in class for hours listening to the professor | 3.5 | 1.12 |
|  | | |
| **Hope Scale statistics: Mean=3.57; SD=0.56; α=0.72** | | |
|  | Mean | SD |
| I am confident when I go to class | 2.83 | 0.86 |
| I am full of hope | 3.34 | 0.93 |
| I am optimistic that I will be able to keep up with the material | 3.90 | 1.02 |
| I am confident because I understand the material | 3.23 | 0.90 |
| Being confident that I will understand the material motivates me | 3.85 | 0.79 |
| My confidence motivates me to prepare for class | 3.53 | 1.06 |
| My hopes that I will be successful motivate me to invest a lot of effort | 4.38 | 0.81 |
|  | | |
| **Pride Scale statistics: Mean=3.84; SD=0.74; α=0.81** | | |
|  | Mean | SD |
| I am proud of myself | 3.57 | 0.91 |
| I take pride in being able to keep up with the material | 3.79 | 1.04 |
| I think that I can be proud of what I know about this subject | 3.88 | 0.97 |
| I am proud of the contributions I have made in class | 3.82 | 0.96 |
| When I make good contributions in class, I get even more motivated | 4.62 | 0.62 |
| Because I take pride in my accomplishments in this course, I am motivated to continue | 4.18 | 0.73 |
| I would like to tell my friends about how well I did in this course | 3.39 | 1.16 |
| When I do well in class, my heart throbs with pride | 4.32 | 1.06 |
|  | | |
| **Anger Scale statistics: Mean=1.29; SD=0.38; α=0.51** | | |
|  | Mean | SD |
| I feel frustrated in class | 2.26 | 1.05 |
| I am angry | 1.18 | 0.65 |
| Thinking about the poor quality of the course makes me angry | 1.08 | 0.27 |
| Thinking about all the useless things I have to learn makes me irritated | 1.23 | 0.82 |
| When I think of the time I waste in class I get aggravated | 1.04 | 0.19 |
| I wish I did not have to attend class because it makes me angry | 1.40 | 0.98 |
| I wish I could tell the teachers off | 1.10 | 0.46 |
|  | | |
| **Anxiety Scale statistics: Mean=2.98; SD=0.77; α=0.86** | | |
|  | Mean | SD |
| Thinking about the class makes me feel uneasy | 3.51 | 1.3 |
| I feel scared | 3.15 | 1.36 |
| I feel nervous in class | 3.47 | 1.16 |
| Even before class, I worry whether I will be able to understand the material | 3.36 | 1.26 |
| I worry whether I am sufficiently prepared for the lesson | 3.91 | 1.01 |
| I worry whether the demands might be too great | 3.50 | 1.04 |
| I worry the others will understand more than me | 2.10 | 1.15 |
| Because I am so nervous I would rather skip the class | 1.29 | 0.77 |
| I get scared that I might say something wrong, so I´d rather not say anything | 2.02 | 0.94 |
| I get tense in class | 3.30 | 1.37 |
| When I do not understand something important in class, my heart races | 3.19 | 1.45 |
|  | | |
| **Shame Scale statistics: Mean=1.97; SD=0.83; α=0.88** | | |
|  | Mean | SD |
| I am ashamed | 2.28 | 1.28 |
| If the others knew that I don´t understand the material I would be embarrassed | 2.66 | 1.45 |
| When I say anything in class I feel like I am making a fool of myself | 1.91 | 1.00 |
| I am embarrassed that I can´t express myself well | 2.40 | 1.36 |
| I am ashamed because others understood more of lecture that I did | 1.33 | 0.76 |
| After I said something in class I wish I could crawl into a hole and hide | 1.44 | 0.74 |
| I´d rather not tell anyone when I don´t understand something in class | 1.76 | 1.15 |
| When I say something in class I feel like I turn red | 2.08 | 1.27 |
| Because I get embarrassed, I become tense and inhibited | 2.21 | 1.25 |
| When I talk in class start stuttering | 1.81 | 1.15 |
|  | | |
| **Hopelessness Scale statistics: Mean=1.22; SD=0.30; α=0.62** | | |
|  | Mean | SD |
| The thought of this class makes me feel hopeless | 1.62 | 0.85 |
| I feel hopeless | 1.28 | 0.66 |
| Even before class, I am resigned to the fact that I won´t understand the material | 1.25 | 0.51 |
| I have lost all hope in understanding this class | 1.06 | 0.23 |
| I feel hopeless continuing in this program of study | 1.20 | 0.49 |
| Because I´ve given up, I don´t have energy to go to class | 1.09 | 0.35 |
| Because I don´t understand the material I look disconnected and resigned | 1.25 | 0.70 |
| I feel so hopeless all my energy is depleted | 1.14 | 0.49 |
|  | | |
| **Boredom Scale statistics: Mean=1.09; SD=0.24; α=0.62** | | |
|  | Mean | SD |
| I get bored | 1.25 | 0.70 |
| Because I get bored my mind begins to wander | 1.38 | 0.81 |
| I am tempted to walk out of the lecture because it is so boring | 1.08 | 0.33 |
| I think about what else I might be doing rather than sitting in this boring class | 1.08 | 0.26 |
| Because the time drags I frequently look at my watch | 1.16 | 0.36 |
| I get so bored I have problems staying alert | 1.00 | 0.00 |
| I get restless because I can´t wait for the class to end | 1.08 | 0.27 |
| During the class I feel like could sink into my chair | 1.02 | 0.14 |
| I start yawing in class because I am so bored | 1.02 | 0.14 |
