# Supplementary material for: Exploring undergraduate students achievement emotions during ward round simulation: a mixed-method study
Source: BMC Med Educ. 2019 Aug 22;19:316. doi: 10.1186/s12909-019-1753-1 (PMC6704623; doi:10.1186/s12909-019-1753-1)
Supplement: Supplementary file 5 — Ward round simulation score (PgWRE) results, with individual components (DOCX 15 kb) [file 12909_2019_1753_MOESM5_ESM.docx]

| Additional file 5: Ward round simulation score (PgWRE) results, with individual components (N=53) |
| --- |

|  | **Score (1-5)*** |
| --- | --- |
| Task management | 4.28 + 0.66 |
| Clinical Skills | 4.19 + 0.73 |
| Acutely ill patients | 4.00 + 0.85 |
| Prescribing techniques | 3.92 + 0.87 |
| Response to interruptions | 4.11 + 0.77 |
| Communication with patients/relatives | 4.58 + 0.63 |
| Communication with colleagues | 4.58 + 0.71 |
| Health and safety | 4.36 + 0.80 |
| Professionalism | 4.26 + 0.65 |
| **Overall PgWRE score** | **4.26 + 0.43** |

*Values expressed as mean + SD
